# A Retrospective Analysis of the Treatment of Cesarean Scar Pregnancy by High-Intensity Focused Ultrasound, Uterine Artery Embolization and Surgery

**DOI:** 10.3389/fsurg.2020.00023

**Published:** 2020-05-27

**Authors:** Shanyu Fang, Ping Zhang, Yuanfang Zhu, Fen Wang, Linsheng He

**Affiliations:** ^1^Department of Obstetrics and Gynecology, The First Affiliated Hospital of Nanchang University, Nanchang, China; ^2^Department of Otorhinolaryngology Head and Neck Surgery, Jiujiang University Hospital, Jiujiang University Clinical Medical College, Jiujiang, China; ^3^Department of Obstetrics and Gynecology, Baoan Maternal and Child Health Hospital, Shenzhen, China

**Keywords:** laparoscopic, hysteroscopy, hysteroscopy–laparoscopic, UAE, HIFU, CSP

## Abstract

**Objective:** This study aims to retrospectively analyze the clinical curative effects of surgery, uterine artery embolization (UAE), and high-intensity focused ultrasound (HIFU) in order to provide the theory and evidences for selecting the optimal treatment for cesarean scar pregnancy (CSP).

**Methods:** Women with CSP were treated with surgery (laparoscopic, hysteroscopy, and hysteroscopy–laparoscopic surgery), UAE combined with curettage, and HIFU combined with curettage. The general conditions and therapeutic effects, including vital signs during the operation, discomfort of discharge, cure rate, total blood loss, decline in the rate of hCG, and hospital stay, were compared and analyzed.

**Results:** For the 154 CSP patients, the cure rate of surgery (*n* = 95) was 97.89%, the cure rate of UAE (*n* = 32) was 43.74%, and the cure rate of HIFU (*n* = 27) was 70.37%. The difference was statistically significant (*P* < 0.05). Furthermore, the hCG level of surgical patients quickly declined, whereas HIFU slowly declined. The difference between the decline rate of hCG and mean hospitalization time was statistically significant (*P* < 0.05). UAE was good for CSP with gestational age <60 days and diameter of gestational sac <40 mm. Furthermore, HIFU was well for CSP patients with a gestational age of <55 days and a gestational sac diameter of <30 mm. Surgery was suitable for any type of these cases.

**Conclusion:** CSP patients with short gestational age and small gestational sac can be treated with surgery, UAE, and HIFU, and achieve safe and effective therapeutic effects. Surgery is also a good choice for CSP for patients with a long gestational age, a large gestational sac diameter, high levels of hCG, or an ample blood supply.

## Introduction

Cesarean scar pregnancy (CSP) is a rare type of ectopic pregnancy, which was first described by Larsen and Solomon (1). At present, due to the prevalence of cesarean section, CSP is no longer a rare event, and its incidence is continuously increasing at a rate of 6.1% for women who had an ectopic pregnancy and at least one cesarean section (2), particularly women in mainland China (3).

Mothers with CSP are confronted with risks, such as unpredictable life-threatening massive bleeding and uterine rupture, when misdiagnosed (4). Hence, there is an urgent need to perform an early accurate diagnosis and select the optimal treatment for CSP, in order to avoid hysterectomy due to uterine rupture and life-threatening massive bleeding (5).

At present, there is no unified standard therapeutic method to cure CSP (6). Conservative treatment entails the systemic or local administration of medication, curettage, and uterine artery embolization (UAE). These methods may lead to uncontrollable massive hemorrhage and hysterectomy. The surgery includes laparotomy, which removes the gestational sacs and previous scars, and hysterectomy. Along with the development and widespread application of minimally invasive surgery, it would be beneficial to treat CSP by laparoscopy and hysteroscopy, since this can reduce surgical trauma and shorten postoperative recovery time (7, 8).

High-intensity focused ultrasound (HIFU) is a non-invasive technique that has properties of safety, effectiveness, precision, no radioactive damage, and less pain. These properties have allowed HIFU to obtain increasing attention from obstetrics and gynecology, which have requirements of retaining the organ and function integrity. It was deduced that HIFU causes a sufficient local rise in temperature that causes necrosis of the gestational sac without damaging the surrounding or overlying tissues, achieving the goal of non-invasive treatment, and avoiding surgical damage (9).

The aim of the present study was to compare and analyze the clinical curative effects of surgery (laparoscopic, hysteroscopy, and hysteroscopy–laparoscopic), UAE combined with curettage, and HIFU combined with curettage in the management of CSP, and provide the theory and evidences for selecting the optimal treatment for CSP.

## Materials and Methods

### Patients

This single-center retrospective study described 154 patients who were diagnosed with CSP in the Department of Obstetrics and Gynecology of the First Affiliated Hospital of Nanchang University from January 2010 to March 2016. Gestational age was calculated based on the last documented menstrual period. These patients were divided into three groups, according to the different treatments: surgery (Group A: A_1_, laparoscopy, 29 patients; A_2_, hysteroscopy, 50 patients; A_3_, hysteroscopy–laparoscopy, 16 patients), UAE (Group B, 32 patients), and HIFU (Group C, 27 patients). A summary of the case series is presented in Table 1. The present study was conducted in accordance with the declaration of Helsinki and was approved by the local ethics committee. A written informed consent was obtained from each participant.

**Table 1:** Comparison of general clinical data among the surgery groups ($\bar{x}$ ± s).

**Group**	**Number of cases**	**Number of pervious cesarean delivery**	**BMI**	**Gestational age (days)**	**Age (years)**	**Interval time (years)**	**Gestational sac diameter (mm)**	**HCG mIu/mL (cases)**	
								**>10,000**	**<10,000**
A_1_	29	1.48 ± 0.68	20.44 ± 2.05	62.29 ± 18.37	31.08 ± 4.94	4.24 ± 1.95	(33.21 ± 18.54) × (22.25 ± 10.46)	16	13
A_2_	50	1.50 ± 0.68	20.63 ± 2.27	51.44 ± 17.08	30.02 ± 4.78	4.15 ± 1.34	(24.08 ± 13.47) × (15.11 ± 11.83)	15	35
A_3_	16	1.63 ± 0.62	20.43 ± 1.31	65.75 ± 23.07	30.92 ± 6.34	4.21 ± 1.10	(32.13 ± 14.31) × (21.88 ± 11.66)	10	6
*P*		>0.05	>0.05	^*^	>0.05	>0.05	^**^	^***^	

*A_1_, laparoscopy group; A_2_, hysteroscopy group; A_3_, hysteroscopy–laparoscopy group*.

*BMI, body mass index*.

* *Comparison between groups A1 and A2, P < 0.05; comparison between groups A1 and A3, P > 0.05; comparison between groups A2 and A3, P < 0.05*.

** *Comparison between groups A1 and A2, P < 0.05; comparison between groups A1 and A3, P > 0.05; comparison between groups A2 and A3, P < 0.05*.

*** *Comparison between groups A1 and A2, P < 0.05; comparison between groups A1 and A3, P > 0.05; comparison between groups A2 and A3, P < 0.05*.

### Diagnosis of CSP

**Figure 1:**
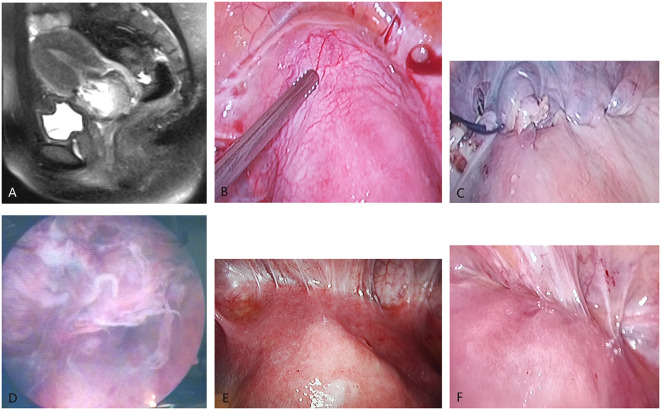
Imaging manifestation of cesarean scar pregnancy (CSP). **(A)** The sagittal MRI shows that the gestational sac attached to the scar of the lower uterine segment. **(B)** The laparoscopy directly shows the CSP lesions (there were abundant blood vessels around uterine scar and hemoperitoneum). **(C)** The appearance of CSP lesions after removal by laparoscopic surgery. **(D)** The appearance of CSP lesions after removal by hysteroscopy. **(E)** The appearance of the uterus in the abdominal cavity under direct vision of laparoscopy in hysteroscopy–laparoscopic surgery (↑ indicates the protuberant CSP lesions). **(F)** The appearance of the uterus after the lesions were removed by hysteroscopy under the direct vision of laparoscopy during the hysteroscopy–laparoscopic surgery (the protuberant CSP lesions disappeared).

(1) Patients with a previous history of lower uterine segment cesarean section. (2) Patients with elevated hCG levels of >5.0 U/L. (3) Patients who underwent an ultrasonographic examination. The diagnostic criteria for the ultrasonographic examination includes the following: (1) visualization of an empty uterine cavity and an empty endocervical canal; (2) detection of a gestational sac in the anterior part of the isthmic portion, and the difference from the endometrial cavity or tubal pregnancy; (3) detection of the gestational sac embedded in the myometrium and fibrous tissue of the cesarean section scar, and absence of the myometrial layer between the gestational sac and bladder; and (4) the presence of a prominent, and at times, rich vascular pattern at or in the area of a cesarean section scar in the presence of a positive pregnancy test. Magnetic resonance imaging (MRI) examination can enhance the diagnostic accuracy of CSP. On sagittal T2-weighted MRI, the gestational sac or products of conception were localized in the previous cesarean section scar, and the depth of placental invasion into the uterine wall was assessed. Based on the degree of placental penetration, the disease condition was subclassified into subtotals. Invasion into the uterine wall during the thinning of the anterior uterine wall was identified, and total invasion into the myometrium during the complete disappearance of the uterine serosal margin was observed (Figure 1A). All patients must match these above criteria.

### Treatment

Clinicians chose the therapeutic method, according to their clinical experience and the specific situation of these patients, such as the diameter of the pregnant bursa, gestational age, blood supply, and hCG level.

#### Group A: Surgery

##### A_1_

The laparoscopic surgery process: The surgeon detected a purplish-blue bulging tissue in the anterior wall of the lower uterine segment. Pituitrin (6 u) that can intensify the constriction of myometrium was dissolved in 20 ml of saline and injected to the bulging tissue. Then, the adhesion from the lower uterine segment and bladder was segregated, the incised tissue of the scar was exposed, and the gestational tissue under the scar was removed. Next, the tissue of the uterine scar was pruned and closed using 1 absorbable suture (Figures 1B,C).

##### A2

Hysteroscopy was used to determine the exact location of the ectopic conceptus and the intrauterine condition in order to distinguish the gestational organization from the uterine isthmus. Then, the sac was removed by diathermy loop excision (Figure 1D). Electrocautery was used when local bleeding was present.

##### A3

The clinicians chose the surgical method described below according to the protrusion of the gestational sac to the uterine. (1) Laparoscopy was used for intraoperative surveillance: The vesicouterine adhesion was exposed by laparoscopy. Hysteroscopy was used to determine the exact location of the ectopic conceptus. Under laparoscopic surveillance, the conceptus was removed by diathermy loop excision through hysteroscopy. The surgeon cleaned up and sucked out the intrauterine hemorrhage, and the uterine surface was observed to have no abnormity. (2) Hysteroscopy was used for intraoperative surveillance: The vesicouterine adhesion was exposed by laparoscopy to visualize the bulge in the lower uterine segment. The surgeon carried out the laparoscopic surgery under hysteroscopic surveillance. It was feasible to block the blood supply of the uterine artery under laparoscopy and subsequently carry out the hysteroscopy surgery for the large gestational sac and lesions with abundant blood supply (Figures 1E,F).

#### Group B: UAE

After the sterilization of the groin, a percutaneous right femoral artery puncture was completed using the Seldinger technique. A 5-F catheter and a guide wire were inserted into the bilateral iliac artery and uterine artery, respectively. Then, digital subtraction arteriography was performed to confirm the location of the bilateral uterine artery and locate the bilateral uterine artery augmentation and circuitous. Both uterine arteries were embolized using gelatin sponge particles. The digital subtraction arteriography was repeatedly conducted to confirm that both uterine arteries were completely embolized. Dilatation and curettage (D&C) was performed after 3 days to remove the conception and blood clots.

#### Group C: HIFU

A JC 200 HIFU system (Chongqing Haifu Technology, Chongqing, China) was used for the ultrasound-guided HIFU, which was equipped with a transducer of 200 mm in diameter and 150 mm in focal length at a frequency of 0.9 MHz and an output power of 350–400 W. During the pretreatment, all patients were required to take liquid food for 3 days. Cleaning enema was performed on the morning of the treatment day after a 12-h fasting period. The patient was laid in the prone position on the treatment table with proper filling of the bladder. The high-intensity ultrasound probe was placed in hypogastric. The skin of the therapy area was placed in the degassing therapy media. The bowel loops in the acoustic pathway were pushed away or compressed by placing a degassed water balloon on the abdominal wall of the patient. The therapeutic target region and layer were set using a concomitant ultrasound imaging device, as a real-time imaging unit, in order to guide the HIFU procedure. The first phase commenced with multiple sonications at ~1–5 s each, which was repeated for 2–10 times, once a day, until the ultrasonic imaging results of the target lesions were enhanced. D&C was performed after 1 day.

### Results Evaluation

Items such as vital signs during the operation, the discomfort of discharge, cure rate, total amount of blood loss (container measurement), the decline rate of hCG, and the length of hospital stay were collected. Standard of effectiveness refers to patients in each group who switched back after treatment and did not require other complementary therapy. Standard of non-effectiveness refers to patients in each group who required other complementary therapies.

### Statistical Analysis

The differences in measurement data among these three groups were presented in ± standard deviation (SD) and compared using single-factor analysis of variance. The difference in enumeration data among these three groups was compared by *X*^2^-test. SPSS 19.0 software (IBM, Armonk, New York, USA) was used, and *P* < 0.05 was considered statistically significant.

## Results

### Group A

#### A1

A total of 29 patients were treated with laparoscopic surgery. The vital signs of these patients were stable, and most of these patients had slight bleeding (~67.62 ml) during surgery. Furthermore, two patients received blood transfusions due to intraoperative massive bleeding (600 and 1,500 ml, respectively). Postoperatively, serum hCG rapidly declined by 13.78–77.3% per day. Most patients had good postoperative recovery and little discomfort at discharge. Merely one patient underwent HIFU complementary therapy, since the sac continued to exist in the scar when rechecked by ultrasound after laparoscopic surgery.

#### A2

A total of 50 patients underwent hysteroscopic surgery. The vital signs of these patients were stable and scarce hemorrhage (~37.56 ml) during the operation. Postoperatively, serum hCG rapidly declined by 29.95–81.36% per day. These patients recovered well without discomfort at discharge. Merely one patient underwent complementary laparoscopy due to persistent vaginal bleeding.

#### A3

A total of 16 patients were treated with hysteroscopy–laparoscopic surgery. The vital signs of these patients were stable but had more hemorrhage (~142.33 ml) without blood transfusions. Postoperatively, serum hCG rapidly declined by 11.68–93.96% per day. Patients recovered well and did not have discomfort at discharge.

### Group B

A total of 32 patients were treated with UAE, and these patients had no peculiar discomfort and bleeding during treatment. D&C was performed after UAE in 14 patients, and these patients had less hemorrhage (~143.03 ml) during D&C. Postoperatively, serum hCG abidingly declined by 8.05–43.56% per day. These patients recovered well and had no discomfort at discharge. However, 5 patients received blood transfusions and laparoscopy or laparotomy due to massive vaginal bleeding after UAE, whereas 13 patients underwent surgery and complementary therapy due to a large gestational sac.

### Group C

A total of 27 patients received outpatient radiation therapy by HIFU for 3–6 times (average: 3.17 times), and all these patients had no discomfort or burn injury during the treatment. Postoperatively, these patients had excessive vaginal bleeding (~176.28 ml). Serum hCG abidingly and gently declined by 2.02–21.68% per day. Eight patients underwent complementary surgery due to massive vaginal bleeding.

### Comparison of General Conditions

According to the statistical analysis, differences in age, cesarean times, body mass index (BMI), and interval time from the previous operation among the three groups (A1, A2, and A3) were not statistically significant (*P* > 0.05). However, differences among the three groups in terms of size of the gestational sac, gestational age, and hCG level before therapy were statistically significant (*P* < 0.05). Moreover, there were no significant differences between the A1 and A3 groups (*P* > 0.05). In addition, the size of the pregnant bursa, gestational age, and hCG of the hysteroscopy group were greater than those of the laparoscopy and hysteroscopy–laparoscopy groups (Table 1).

There is no significant differences in age, times of cesarean section, BMI, and interval time from the previous operation among groups A, B, and C (*P* > 0.05). However, differences among these three groups in terms of size of the gestational sac, gestational age, and hCG level before therapy were statistically significant (*P* < 0.05). Furthermore, the differences between groups A and C were not statistically significant (*P* > 0.05). Moreover, the size of the pregnant bursa, gestational age, and hCG were greater in the UAE group than in the surgery and HIFU groups (Table 2).

**Table 2:** Comparison of general clinical data among the three groups ($\bar{x}$ ± s).

**Group**	**Number of cases**	**Number of previous cesarean delivery**	**BMI**	**Gestational age (days)**	**Age (years)**	**Interval time (years)**	**Gestational sac diameter (mm)**	**HCG mIu/mL (cases)**	
								**>10,000**	**<10,000**
A	95	1.52 ± 0.66	20.54 ± 2.07	57.23 ± 19.41	30.49 ± 4.95	4.19 ± 1.48	(28.77 ± 15.96) × (18.87 ± 11.74)	41	54
B	32	1.59 ± 0.79	20.40 ± 1.91	68.05 ± 23.29	30.39 ± 5.78	3.81 ± 2.64	(32.28 ± 15.95) × (20.96 ± 15.68)	22	10
C	27	1.37 ± 0.63	20.49 ± 2.01	51.08 ± 13.47	30.55 ± 4.01	4.91 ± 2.58	(24.35 ± 10.22) × (15.84 ± 8.51)	7	20
*P*		>0.05	>0.05	<0.05^*^	>0.05	>0.05	^**^	^***^	

*A, surgery group; B, UAE group; C, HIFU group*.

*BMI, body mass index*.

* *Comparison between every two groups in groups A, B, and C, P <0.05*.

** *Comparison between group A and group B, P > 0.05; comparison between group A and group C, P > 0.05; comparison between group B and group C, P <0.05*.

*** *Comparison between group A and group B, P <0.05; comparison between group A and group C, P > 0.05; comparison between group B and group C, P <0.05*.

### Comparison of Therapeutic Effects

The differences in total curative effects among the three surgery groups (A1, A2, and A3) were not statistically significant (*P* > 0.05, Table 3). This shows that hospitalization time was significantly shorter in group A2 than in the other two groups (A1 and A3; *P* < 0.05). Furthermore, the amount of bleeding was significantly greater in group A3 than in the other two groups (*P* < 0.05). Moreover, bleeding volume was significantly greater in group A3 than in the other two groups (*P* < 0.05).

**Table 3:** Compared postoperative situation of each surgery group.

**Group**	**General condition**	**Amount of bleeding**		**Hospital stays (days)**	**Total curative effects**		
		**<100 ml (cases)**	**>100 ml (cases)**		**Cure (cases)**	**Failure (cases)**	**First cured rate (%)**
A_1_	Without discomfort	26	3	7.27 ± 2.20	28	1	96.55
A_2_	Without discomfort	48	2	5.79 ± 2.01	49	1	98.00
A_3_	Without discomfort	5	11	7.31 ± 2.15	16	0	100.00

*A_1_, laparoscopy group; A_2_, hysteroscopy group; A_3_, hysteroscopy–laparoscopy group*.

**Figure 2:**
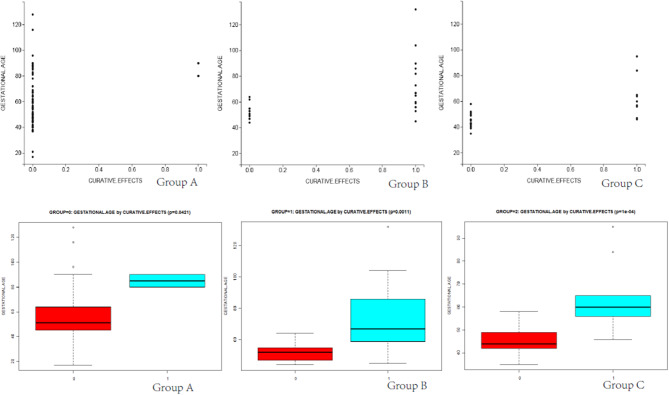
The scatter diagram and box plots of the relationship between gestational age and curative effects in groups A, B, and C are shown. The red box represents successful and green box represents failed.

**Figure 3:**
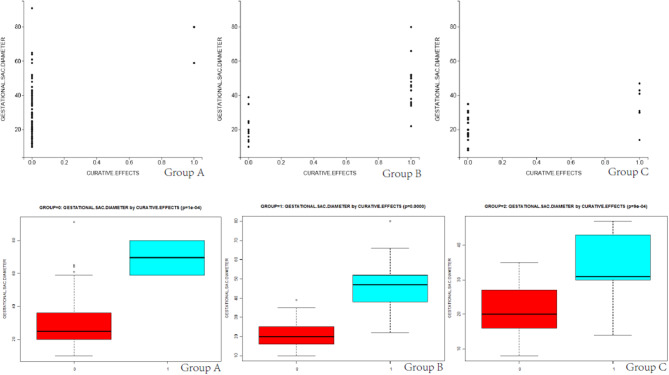
The scatter diagram and box plots of the relationship between the size of the gestational sac and curative effects in groups A, B, and C are shown.

Differences in total blood loss, the decline rate of HCG, and hospital stay among groups A, B, and C (Table 4) were statistically significant (*P* < 0.05). In group A, the average blood loss was 99.07 ml, the decline of hCG was 45.43% per day, and the mean hospitalization time was 6.35 days. There was no hemorrhage during the process of UAE and HIFU treatment, but excessive hemorrhage occurred after subsequent curettage. In group B, the average blood loss was 143.03 ml, the decline rate of hCG was 17.68% per day, and the mean hospitalization time was 8.53 days. After treatment by HIFU, the average blood loss was 176.28 ml. Furthermore, hCG exhibited a slow rate of decline, with an average decline rate of 7.81% per day. These patients were directly cured by outpatient HIFU without hospitalization. The cured rate of surgery was 97.89% (UAE was 43.74% and HIFU was 70.37%), and the differences were statistically significant (*P* < 0.05). As shown in Figures 2, 3, in the group of UAE, when gestational age was <60 days, 12 patients were cured and 3 patients were not cured. When gestational age was more than 60 days, 15 patients were not cured, whereas only 2 patients were cured. When gestational sac diameter was <30 mm, 11 patients were cured and 1 patient was not cured. When gestational sac diameter was more than 30 mm, 17 patients were not cured and only 3 patients were cured. In the group of HIFU, when gestational age was <55 days, 15 patients were cured and 3 patients were not cured. When gestational age was more than 55 days, 6 patients were not cured, whereas only 3 patients were cured. When the gestational sac diameter was <30 mm, 15 patients were cured and 4 patients were not cured. When the gestational sac diameter was more than 30 mm, 5 patients were not cured and only 3 patients were cured. Hence, UAE was good for CSP with a gestational age of <60 days and a gestational sac diameter of <40 mm. Furthermore, HIFU was well for CSP patients with a gestational age of <55 days and a gestational sac diameter of <30 mm. Surgery was suitable for any type of these cases.

**Table 4:** Comparison of postoperative situation among the three groups.

**Group**	**General condition**	**Amount of bleeding**			**Hospital stays (days)**	**Decline rate of hCG every day (%)**	**Total curative effects**		
		**<100 ml (cases)**	**>100 ml (cases)**	**Average**			**Cure (cases)**	**Failure (cases)**	**First cured rate (%)**
A	Without discomfort	79	16	99.07	6.53 ± 2.21	45.43 ± 22.61	93	2	97.89
B	Without discomfort	6	26	143.03	8.53 ± 1.91	17.68 ± 5.28	14	18	43.75
C	Without discomfort	3	24	176.28	-	7.81 ± 1.19	19	8	70.37

*A, surgery group; B, UAE group; C, HIFU group*.

## Discussion

CSP is the long-term complication of cesarean section. Although the mechanism of CSP remains uncertain, it is possible that previous cesarean section, multiple curettage, or endometriosis could lead to microscopic defects or dehiscent tract in the scar due to poor recovery. Contrast-enhanced ultrasound has a higher accuracy than conventional ultrasound in the diagnosis of CSP (10). Medical treatment entails medication, curettage, UAE, and surgery. However, to date, no optimal CSP therapy has been reported.

Laparoscopy and hysteroscopy have the properties of minimal trauma and rapid recovery. It is an effective treatment to completely eliminate the gestational tissue, with appropriate repair and suture dehiscence of the uterine scar (11). Blocking of both uterine arteries can be performed to reduce hemorrhage too. Furthermore, injected pituitrin can be opted at the same time, according to the specific situation of the lesion (12). Hysteroscopy can further decrease surgical trauma and abridge recovery time, according to the natural orifice for females, and it can remedy the disadvantages of blind curettage. It is an effective therapeutic method for CSP (13). Research has shown that hysteroscopy is suitable for patients whose gestational sac protrude to the uterine cavity (14). However, laparoscopy is suitable for the cases where the gestational sac is protruding to the abdominal cavity (15). Hysteroscopy–laparoscopic surgery has the combined advantages of both hysteroscopy and laparoscopy, and despite being costly, it is the ideal method of treatment for patients who cannot be treated using only one endoscopic method (16). In the present study, the curative effects were best among the three surgical groups. The period of hospitalization was shortest. Merely two patients required other complementary treatments. The reason may be that the gestational sacs were large and could not be completely removed.

As a non-invasive therapy, recent studies have demonstrated that UAE can block uterine artery blood flow, which results in trophoblastic cell degeneration and necrosis (17). UAE, in combination with drugs or curettage, can achieve better curative effects (18, 19). A study revealed that the gestational age of CSP is >8 weeks, and a CSP mass of ≥6 cm tends to have an unsatisfactory outcome after UAE, followed by curettage (20). However, for patients who have massive vaginal bleeding and high operation risk, UAE can be performed before surgical treatment in order to reduce hemorrhage and improve the safety of the operation. In the present study, some patients who received UAE, followed by curettage, rapidly recovered. Concretely speaking, hemorrhage was <100 ml, hospitalization was ~4 days, and hCG declined by 43.56% per day. Nevertheless, most patients required other methods of complementary treatment. This was probably due to the long gestational age, which was mostly more than 60 days, a sac dimension of >40 mm, high levels of hCG, and uterine arteries with collateral circulation.

Recently, HIFU has gained much attention, and several clinical applications have been reported over the course of the previous years, even in the field of gynecology, and for the treatment of uterine fibroids and adenomyosis (21–25). By focusing beams of ultrasound energy to CSP lesions, HIFU can accumulate high intensities in targeted tissues, which eventually result in complete coagulation necrosis, and the surrounding normal tissues that almost do not receive any damage (8). The research led by Zhu et al. (26) indicated that HIFU is safe and effective for treating CSP patients at gestational ages of <8 weeks. In the present study, some patients received HIFU, followed by curettage. Hemorrhage was <100 ml, and hCG declined by 21.68% per day. Some patients required complementary treatment due to colporrhagia and the persistent existence of a gestational sac. The reason may be that these patients had a long gestational age of >55 days and a large gestational sac diameter of >30 mm, which lead to failure of HIFU. HIFU does not require anesthesia. It is non-invasive, is safe, and does not require hospitalization, which is worthy of clinical promotion.

## Conclusion

The present study suggests that CSP patients with short days of gestational age and a small gestational sac can be treated with surgery, UAE, and HIFU, and achieve safe and effective therapeutic effects. Surgery is a good choice for CSP patients, especially for patients with a long gestational age, a large gestational sac diameter, high levels of hCG, or an ample blood supply. Otherwise, the risk of hemorrhage would increase. Hence, HIFU or UAE can be considered as an adjuvant therapy before laparoscopy, hysteroscopy, or hysteroscopy–laparoscopic in order to reduce intraoperative bleeding and increase the safety of the surgery. Because this study was a retrospective analysis, the standard treatment for CSP to select still needs further perspective study.

## Data Availability Statement

All datasets generated for this study are included in the article/supplementary material.

## Ethics Statement

This study was carried out in accordance with the recommendations of Ethics Committee of the First Affiliated Hospital of Nanchang University with written informed consent from all subjects. All subjects gave written informed consent in accordance with the Declaration of Helsinki. The protocol was approved by the First Affiliated Hospital of Nanchang University. The patients/participants provided their written informed consent to participate in this study.

## Author Contributions

SF collected and analyzed the data and wrote the manuscript. PZ collected and analyzed the data. YZ designed the study. FW analyzed the data and revised the manuscript. LH designed the study and revised the manuscript.

## Conflict of Interest

The authors declare that the research was conducted in the absence of any commercial or financial relationships that could be construed as a potential conflict of interest.

## Abbreviations

CSP: cesarean scar pregnancy
D&C: dilatation and curettage
HIFU: high-intensity focused ultrasound
SD: standard deviation
UAE: uterine artery embolization.
